# Probing Critical Physical Properties of Lactose-Polyethylene Glycol Microparticles in Pulmonary Delivery of Chitosan Nanoparticles

**DOI:** 10.3390/pharmaceutics13101581

**Published:** 2021-09-29

**Authors:** Nasser Alhajj, Idanawati Naharudin, Paolo Colombo, Eride Quarta, Tin Wui Wong

**Affiliations:** 1 Non-Destructive Biomedical and Pharmaceutical Research Centre, Smart Manufacturing Research Institute, Universiti Teknologi MARA Selangor, Puncak Alam 42300, Selangor, Malaysia; nasserph@hotmail.com (N.A.); idana482@uitm.edu.my (I.N.); 2 Particle Design Research Group, Faculty of Pharmacy, Universiti Teknologi MARA Selangor, Puncak Alam 42300, Selangor, Malaysia; 3 PlumeStars srl, 43125 Parma, Italy; 4 Food and Drug Department, University of Parma, 43121 Parma, Italy; eride.quarta@studenti.unipr.it; 5 Sino-Malaysia Molecular Oncology and Traditional Chinese Medicine Delivery Joint Research Centre, Medical College, Yangzhou University, 136, Jiangyang Middle Road, Yangzhou 225001, China

**Keywords:** chitosan, lactose, microparticle, nanoparticle, pulmonary delivery

## Abstract

Pulmonary delivery of chitosan nanoparticles is met with nanoparticle agglomeration and exhalation. Admixing lactose-based microparticles (surface area-weighted diameter~5 μm) with nanoparticles mutually reduces particle agglomeration through surface adsorption phenomenon. Lactose-polyethylene glycol (PEG) microparticles with different sizes, morphologies and crystallinities were prepared by a spray drying method using varying PEG molecular weights and ethanol contents. The chitosan nanoparticles were similarly prepared. In vitro inhalation performance and peripheral lung deposition of chitosan nanoparticles were enhanced through co-blending with larger lactose-PEG microparticles with reduced specific surface area. These microparticles had reduced inter-microparticle interaction, thereby promoting microparticle–nanoparticle interaction and facilitating nanoparticles flow into peripheral lung.

## 1. Introduction

Lactose is widely used in inhalation dosage form development with reference to dry powder aerosol [1,2,3,4]. It is safe, and exhibits good physicochemical stability and powder flow properties [5]. Lactose, mannitol, sorbitol and dextran are inhalational sugar and polyalcohol carriers for drugs [6], but the only approved carrier is lactose [4].

The United States Food and Drug Administration indicates that a nanoproduct has at least one nanoscale dimension (about 1 nm to 100 nm), or exhibits physicochemical or biological properties attributable to its nanogeometry, up to one µm [7]. The nanocarrier presents several advantages in pulmonary dosage form design: (I) the nanocarrier has a large specific surface area to interact with the lung; (II) cell- and organelle-targeting can be elicited by decorating nanocarrier with targeting ligand; and (III) the nanocarrier is characterized by a higher lung cell uptake, leading to reduced required drug dosage and side effects [8,9].

Chitosan is widely explored as a pulmonary therapeutic carrier in lung cancer therapy [10,11,12,13,14,15,16]. The pulmonary chitosan carrier brings about reduced systemic drug toxicity, increased lung drug bioavailability and improved anti-cancer drug efficacy. The cellular uptake of engineered particles is improved and cancer cell apoptosis is promoted [10,11,12,13,14,15,16].

Chitosan nano- and micro-carriers have been developed as pulmonary drug vehicles [15,17,18]. The suitability of chitosan solid nanoparticles and liquid nanoemulsion for pulmonary drug delivery has been examined. The solid nanoparticles are prone to exhalation where particles less than 1 µm can be exhaled up to 80% after inspiration without being deposited, unlike particles with an aerodynamic diameter between 1 and 3 µm, which can be delivered into the lower lung [19,20]. They are highly aggregative with inefficient dispersion and inhalation performances [19,20]. The solid nanoparticles have been co-spray dried with leucine, lactose, maltodextrin or mannitol into microparticles or physically admixed with lactose-polyethylene glycol 3000 microparticles to promote their inhalation efficiency [20,21,22]. The co-spray dried microparticles are small (mass median aerodynamic diameter = 3–10 µm) [22]. They are, however, aggregative and not desirable for pulmonary inhalation [5,23]. Blending of co-spray dried microparticles with large carriers (63–90 µm) improves microparticle dispersibility [5]. The large carrier carries the microparticles through surface adsorption. The microparticles were detached from the large carrier during the inhalation process and were deposited in the lung [24].

A direct chitosan nanoparticles-lactose-polyethylene glycol 3000 microparticle blend enhances the powder flow, similar to the microparticles-large carriers mix [20,24,25]. Microparticles as well as nanoparticles are cohesive. Dispersing the nanoparticles over the microparticles’ surfaces through mixing in a specific weight ratio promotes the dispersibility of nanoparticles [26]. The adsorbed nanoparticles are functionally a glidant. They can reduce the aggregation of microparticles to produce an inhalable powder.

Unlike co-spray drying, direct chitosan nanoparticles-lactose based microparticle blending reduces the likelihood of changes in nanoparticulate properties [20,27,28]. The size-dependent biological responses of nanoparticles are expected to deviate to a lesser extent by blending than microencapsulation by co-spray drying [20,27,28]. The surface-deposited nanoparticles are re-dispersible. They can detach more readily from the microparticle surfaces and are inhaled into the lung with a reduced entanglement tendency as per the microencapsulated system.

The size and shape, as well as specific surface area, of nanoparticles, admixed with lactose-based microparticles, affect their inhalation efficiency [20]. Small and irregularly shaped nanoparticles have a large specific surface area and are aggregative. They are not deposited onto the surfaces of microparticles and experience a low inhalation efficiency. Over the past few decades, the majority of inhalation studies focus to identify the critical physicochemical attributes of large carriers for pulmonary delivery of microparticles (3 µm to 10 µm). There is no known study evaluating the critical design of microparticles as the pulmonary carrier of nanoparticles. The latter represents a greater challenge as both nanoparticles and microparticles are cohesive. Both nanoparticles and microparticles are relatively cohesive in that they are small in size and are characterized by a large inter-particulate-specific surface area. To enable nanoparticles to adsorb on the microparticulate surfaces and to transform into an inhalable formulation, it is hypothesized that larger, rounder and smoother microparticles are required to enable nanoparticles to disaggregate and adsorb onto the microparticulate surfaces without developing a cohesive blend. As such, this study assesses the inhalation performances of chitosan nanoparticles-lactose-polyethylene glycol microparticle blends using microparticles developed with different physicochemical characteristics. The study aims to identify the critical properties of lactose-polyethylene glycol microparticles that are required to deliver the chitosan nanoparticles by inhalation means.

## 2. Materials and Methods

### 2.1. Materials

Lactose monohydrate (Sorbolac 400, Meggle, Wasserburg, Germany) was used as the matrix material of microparticles. Polyethylene glycol 400, 1000 and 3000 (PEG 400, PEG 1000, PEG 3000; Merck, Darmstadt, Germany) were used as the stabilizers with ethanol absolute (Merck, Germany) as the additive. Chitosan (molecular weight: 20,000–50,000 g/mol, degree of deacetylation ≥ 90%; Zhejiang Aoxing Biotechnology Co. Ltd., Zhejiang, China) was used as the model matrix material that was transformable into nanoparticles, with glacial acetic acid as a solvent (Merck, Germany). Lithium acetate anhydrous (ACROS Organics™, Waltham, MA, USA), ninhydrin and hydrindantin (Sigma-Aldrich, Saint Louis, MO, USA) were the reagents used for the quantitative assay of chitosan.

### 2.2. Preparation of Lactose-PEG Microparticles

Lactose microparticles L1-6 were prepared by a spray drying technique (Table 1; TwinNanoSpray, UiTM, Selangor, Malaysia). The lactose was first dissolved in the distilled water. It was then added with 2.5 %w/w PEG, with reference to dry lactose weight, at 25 ± 1 °C. When applicable, the ethanol was introduced as the co-solvent of lactose and PEG. PEGs were used as the stabilizer of lactose microparticles. Their molecular weights ranged from 400 to 3000 g/mol.

The spray-dried powder was collected by a precipitating electrode. It was kept in a 30-mL glass jar. The powder was stored in a desiccator at 25 ± 1 °C for 5 days prior to characterization.

### 2.3. Preparation of Chitosan Nanoparticles

Chitosan (0.1 %w/w) was dissolved in 0.5 %v/v acetic acid under continuous stirring for 5 h at 25 ± 1 °C. The solution was subjected to nanospray-drying (TwinNanoSpray, UiTM, Malaysia) with inlet temperature = 70 °C, outlet temperature = 24.8 °C, solution feed rate = 3 mL/min via bifeeding tubes, and atomizing air pressure = 6 bar. The spray-dried powder was collected at the precipitating electrode. It was retrieved into a 10 mL clear glass powder jar by a rubber spatula. The powder was stored in a desiccator at 25 ± 1 °C prior to characterization.

### 2.4. Physicochemical Analysis of Lactose-PEG Microparticles

#### 2.4.1. Density

A known weight of powder was added in a 5-mL measuring cylinder. The quotient of powder weight to bulk volume was recorded as the bulk density (*ρb*). The cylinder was subjected to 200 taps, where there was no further powder bed volume reduction thereafter, at 4 taps/s. The tapped density (*ρt*) was recorded as the quotient of powder weight to its tapped volume. Both bulk density and tapped density values of powder were used to derive Carr’s index and Hausner ratio by means of the following equations:

Carr’s index = (1 − *ρb/ρt*) × 100% (1)

Hausner ratio = *ρt*/*ρb* (2)

#### 2.4.2. Size

The size distribution of microparticles was determined using the laser diffraction analyzer (Mastersizer 2000, Malvern Instruments Ltd., Worcestershire, UK). The volume weighted mean diameter (D_[4,3]_), surface area weighted mean diameter (D_[3,2]_), specific surface area, span, volume median diameter d_10_, *d*_50_ and *d*_90_ were assessed by means of dry powder dispersion mode (Scirocco 2000) at 1 bar pressure. Triplicates were run for each type of microparticles with the results averaged.

#### 2.4.3. Scanning Electron Microscopy

The surface morphology of microparticles was evaluated by scanning electron microscope (SEM) (Quanta 450 FEG, FEI, Eindhoven, The Netherlands). The microparticles were adhered onto a carbon tape, and sputter-coated with 5 nm-thick platinum with an auto fine coater (JFC-1600, JEOL, Akishima, Japan). The surface roughness (R_a_) and circularity (Circ) of microparticles were analyzed by ImageJ software (NiH, Bethesda, MD, USA). A minimum of nine measurements of three images were characterized.

#### 2.4.4. X-ray Powder Diffraction

The crystallinity of microparticles was examined by the X-ray powder diffractometer (XRPD) (Ultima IV, Rigaku Coperation, Akishima, Japan), over diffraction angles (2*θ*) of 3° to 60° at 5°/min scanning speed. Cu-Kα (40 kV and 30 mA) was used as the X-ray resource. Triplicates were conducted and the results were averaged.

### 2.5. Physicochemical Characterization of Chitosan Nanoparticles

The surface morphology and crystallinity of the chitosan nanoparticles were examined by SEM and XRPD techniques respectively. The chitosan crystallinity index (cCI; %) was calculated as:

(3) $$\mathrm{cCI}=\frac{(I_{110}-I_{\mathrm{amor}})\times 100}{I_{110}}$$

where I_110_ = maximum intensity at 20° and I_amor_ = intensity of amorphous diffraction at 16° [29,30].

#### 2.5.1. Size

The size and polydispersity index were determined using the Zetasizer (Nano ZS 90, Malvern Instruments Ltd., Worcestershire, UK) at 25 ± 1 °C and 90° scattering angle. The nanoparticles (5 mg) were dispersed in deionized water (30 mL) with the aid of magnetic stirring. Triplicates were determined with results averaged.

#### 2.5.2. Zeta Potential

The zeta potential was determined using the Zetasizer (Nano ZS 90, Malvern Instruments Ltd., Worcestershire, UK) at 25 ± 1 °C. The nanoparticles (5 mg) were dispersed in deionized water (30 mL) with the aid of magnetic stirring. Three measurements were determined with results averaged.

### 2.6. In Vitro Aerosolization and Inhalation

Andersen Cascade Impactor (Copley Scientific Ltd., Nottingham, UK) was used to assess the aerosolization and inhalation profiles of nanoparticles as a function of physicochemical characteristics of lactose-PEG microparticles. The stage F-0 were assembled with silicone rubber O-rings to avoid leakage from gaps between stages. A glass fiber filter (Copley Scientific Ltd., Nottingham, UK) was placed beneath stage 7 to capture small particles from upper stages. A pre-separator was attached to stage 0 with a connecting induction port assembled with mouthpiece adapter and Handihaler^®^ (Boehringer Ingelheim, Ingelheim am Rhein, Germany). The impactor was equipped with a vacuum source (LCP5, Copley Scientific Ltd., Nottingham, UK) and a critical flow controller (TPK 2000, Copley Scientific Ltd., Nottingham, UK).

The chitosan nanoparticles and lactose-PEG microparticles (weight ratio 1:9) were blended with 40 Hz vortex for 30 min (VelpScientifica, Usmate, Italy). Thirty mg of powder mixture were inserted into a size-2 capsule (San Tronic Medical Devices, Selangor, Malaysia). The capsule was inserted into the chamber of Handihaler^®^. The powder in the inhaler was actuated to the impactor through an air current flow of 48 L/min for 5 s which corresponded to a 4 kPa pressure drop as in human lung. The cut-off diameter of each stage was calculated using Equation (4): Stage 0, 6.91 µm; Stage 1, 4.45 µm; Stage 2, 3.61 µm; Stage 3, 2.53 µm; Stage 4, 1.61 µm; Stage 5, 0.84 µm; Stage 6, 0.54 µm; and Stage 7, 0.31 µm.

(4) $$D_{50,48}=D_{50,28.3}{\left(\frac{28.3}{48}\right)}^{\frac{1}{2}}$$

where D_50,48_ and D_50,28.3_ are the cut-off diameters at 48 and 28.3 L/min respectively.

The contents of 5 capsules (15 mg chitosan nanoparticles) were released into the impactor sequentially in a cumulative fashion. Triplicates were conducted, and with impactor cleaned before another 5 capsules were tested. The emitted powders on mouthpiece, induction port, pre-separator, collection plate and filter were collected using 0.5 %v/v acetic acid solution. The samples were stored in individual glass vials, oscillated in a shaker bath (ST402, Nuve, Ankara, Turkey) at 25 ± 1 °C for 5 h, and had chitosan content determined by ninhydrin assay method. The chitosan content was assayed using UV-VIS spectrophotometer (Cary 50 Conc, Varian Australia Pty. Ltd., Mulgrave, Australia) at 570 nm wavelength. The detection and quantification limits were 0.048 mg/mL and 0.160 mg/mL respectively. The linearity ranged between 0.025 and 1.000 mg/mL.

The emitted dose (ED) referred to the collective chitosan mass on mouthpiece, induction port, pre-separator and all stages. Deposited dose (DD) referred to chitosan mass on stages 0 to F. Percent dispersed (PD) was defined as percentage ED in relation to total dose (TD). Percent inhaled (PI) was defined as percentage DD in relation to TD. The fine particle doses (FPD), FPD_<4.5µm_, FPD_<3.6µm_, and _0.5µm<_FPD_<3.6µm_, were doses on stages 2 to F, stages 3 to F, and stages 3 to 7 respectively. FPD_<4.5µm_ reflected deep lung-deposited particles. FPD_<3.6µm_ reflected peripheral lung-deposited particles. _0.5µm<_FPD_<3.6µm_ represented FPD_<3.6µm_ excluding particles prone to exhalation (<500 nm). The fine particle fraction (FPF) was defined as percentage FPD to ED. The respirable fraction (RF) was denoted by percentage of FPD to DD.

The cumulative particle size distribution pattern was plotted on a log probability graph. The particle size at 50th percentile of distribution was denoted as mass median aerodynamic diameter (MMAD). The square root of the ratio of particle size at 84.13th percentile to 15.87th percentile was denoted as a geometric standard deviation (GSD). Triplicates were run for each experiment with the results averaged.

### 2.7. Fourier Transform Infrared (FTIR) Spectroscopy

The segregation pattern of chitosan nanoparticles from lactose-PEG microparticles upon inhalation was characterized. L3 and L6 admixed with chitosan nanoparticles were collected from stages 0 to 7. The powder mixture was blended with potassium bromide at a 1:99 weight ratio and was subjected to grinding. The ground mixture was compressed by an axial load (Specac Ltd., Kent, UK) to a maximum pressure of 10 tons for 2 min. The FTIR spectrum of the formed disc was obtained by an IR spectrometer (Spectrum 100, Perkin Elmer, Richmond, UK) through scanning over 400 to 4000 cm^−1^ and at 4 cm^−1^ resolution. Chitosan nanoparticles, lactose-PEG microparticles and the mixture of both (weight ratio 1:9) were used as the references. The correlation of FTIR spectra between impactor powder samples and references was analyzed using Perkin Elmer Spectrum Version 10.3.6 software (Perkin Elmer, Richmond, UK) by means of the following equation [20]:

(5) $$\mathrm{Correlation}\;\mathrm{coefficient}=\frac{\sum w_{i}\;A_{i\;}B_{i}}{{\left(\sum w_{i}\;A_{i\;}A_{i}\right)}^{0.5}\times {\left(\sum w_{i}\;B_{i\;}B_{i}\right)}^{0.5}}$$

where *A_i_* = spectra absorbance value of test powder at frequency *i*, *B_i_* = spectra absorbance value of reference powder at frequency *i*, and *w_i_* = a statistical weighting factor. Triplicates of experiment were conducted with results averaged.

## 3. Results and Discussion

### 3.1. Physicochemical Characteristics of Lactose-PEG Microparticles

The physicochemical characteristics of six lactose-PEG microparticle variants are summarized in Table 2.

#### 3.1.1. Size

Lactose-PEG microparticles were characterized by *d*_50_ ranging from 5.43 ± 0.10 µm (L3) to 13.32 ± 0.15 µm (L5) (Table 2). PEG 3000 had a lower affinity for water than PEG 400 and PEG 1000 [31]. With reference to L3, it was envisaged that the spray droplets were small due to reduced PEG-water tension. The dried particles were thus accordingly small.

Lactose is soluble in water (0.22 g/mL at 25 °C). It is practically ethanol-insoluble. PEG is soluble in ethanol, with a larger molecular weight PEG having a higher solubility [31]. In the case of L6, the lactose was envisaged to accumulate in the aqueous phase. PEG 3000 was attracted to the organic phase. Larger spray droplets were anticipated due to reduced contact of lactose-PEG. The size of spray-dried particles in the presence of ethanol was larger than in the ethanol-free sample (L3). The effect of ethanol on particle size was not apparent with PEG 400 and PEG 1000. Being lower in molecular weight, these PEGs had a higher affinity to water than ethanol. The formation of spray-dried particles was less affected by ethanol.

#### 3.1.2. Morphology

**Figure 1:**
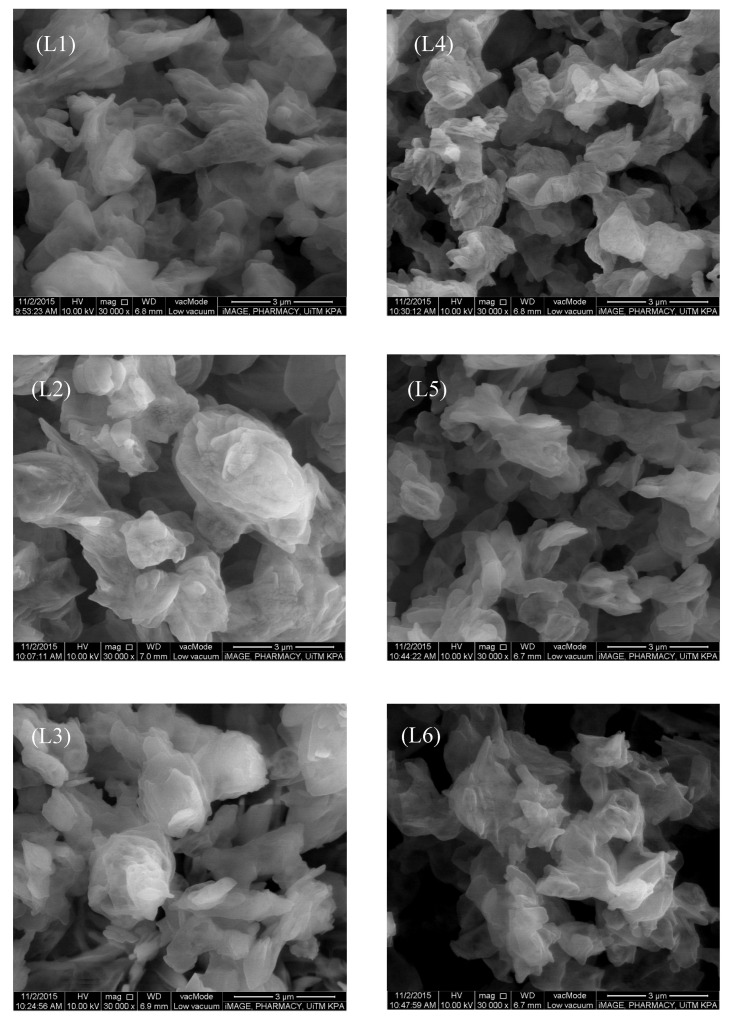
Scanning electron microscopic images of lactose-PEG microparticles.

All batches of lactose-PEG microparticles were irregular in shape with some variations (Figure 1; Table 2). In terms of surface roughness, L2 and L5 that were formulated with PEG 1000 had a noticeable lower Ra values (Table 2). PEG 400 had a lower tackiness than PEG 1000. Its use might be accompanied by irregular contact with the lactose due to poor attachment property. In the case of PEG 3000, the higher viscosity PEG was envisaged to exhibit a poor spread over lactose. The summative effect was that PEG with molecular weight higher or lower than 1000 g/mol brought about the formation of lactose-PEG microparticles with rougher surfaces.

#### 3.1.3. Crystallinity

The spray-dried lactose microparticles were hygroscopic and experienced a marked aggregation during handling. PEG could transform the spray-dried lactose with a higher crystallinity level. It could form hydrogen bonds with water in the course of spray drying. The PEG could delay the lactose drying and provide more time for lactose molecules to rearrange into a crystalline structure [32,33].

**Figure 2:**
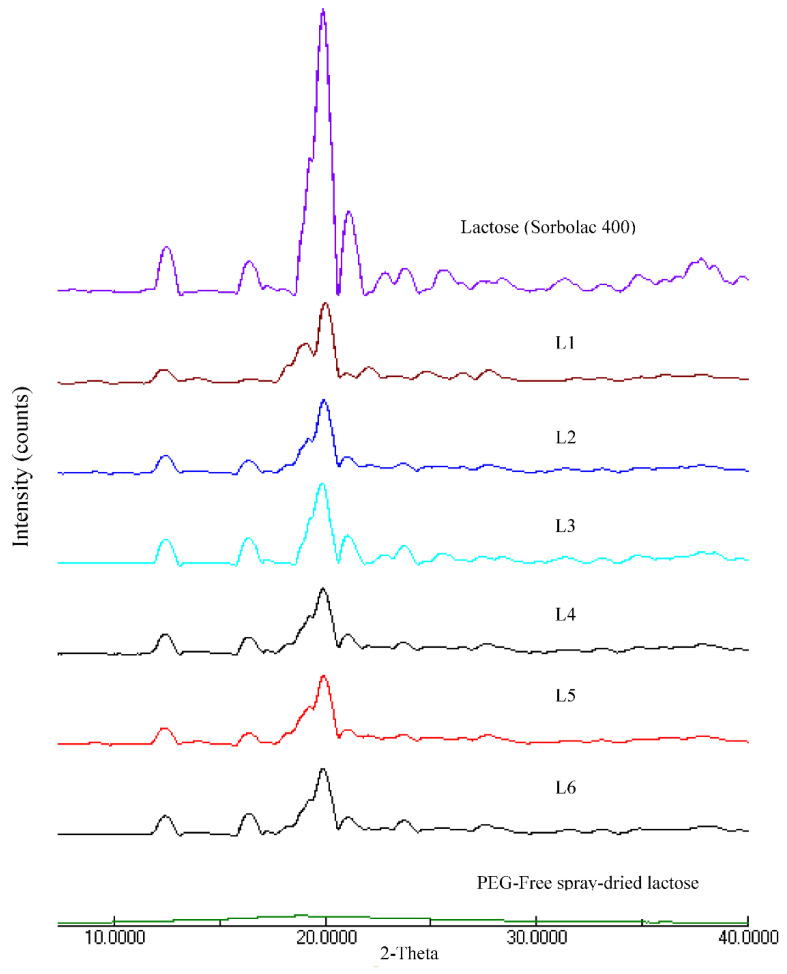
X-ray powder diffractograms of lactose and lactose-PEG microparticles.

The crystallinity of L1 to L6 microparticles were lower than unprocessed lactose (peak area_2*θ=*19.98°_ = 1640.67 ± 75.13) and higher than PEG-free spray-dried lactose (peak area_2*θ=*19.98°_ = 15.85 ± 7.14) (Table 2; Figure 2). In the case of L1 and L4, the ethanol-free formulation exhibited a higher matrix crystallinity. Using ethanol as an additive, the drying rate of spray droplets was anticipated to be fast and resulted in the formation of more amorphous lactose-PEG microparticles. Lower molecular weight PEG is less interactive with ethanol [31]. The speed of drying droplets with a lower molecular weight PEG can be promoted by ethanol to an extent where matrix crystallinity was reduced to a noticeable degree.

The ethanol-free formulations demonstrated a decrease in matrix crystallinity with increasing PEG molecular weight (Table 2). The water affinity of PEG decreases with an increase in PEG molecular weight. Using lower molecular weight PEG, the water evaporated at a slower rate and allowed lactose to rearrange into a more crystalline structure in the form of dried particles. The introduction of ethanol in L2 and L3 resulted in an increase in matrix crystallinity, unlike L1 (Table 2). Lactose particles in aqueous solution tend to solidify as amorphous lactose [34]. The addition of ethanol into the feed solution might decrease lactose solubility, which in turn reduces the solidified amorphous lactose [35].

### 3.2. Physicochemical Characteristics of Chitosan Nanoparticles

**Figure 3:**
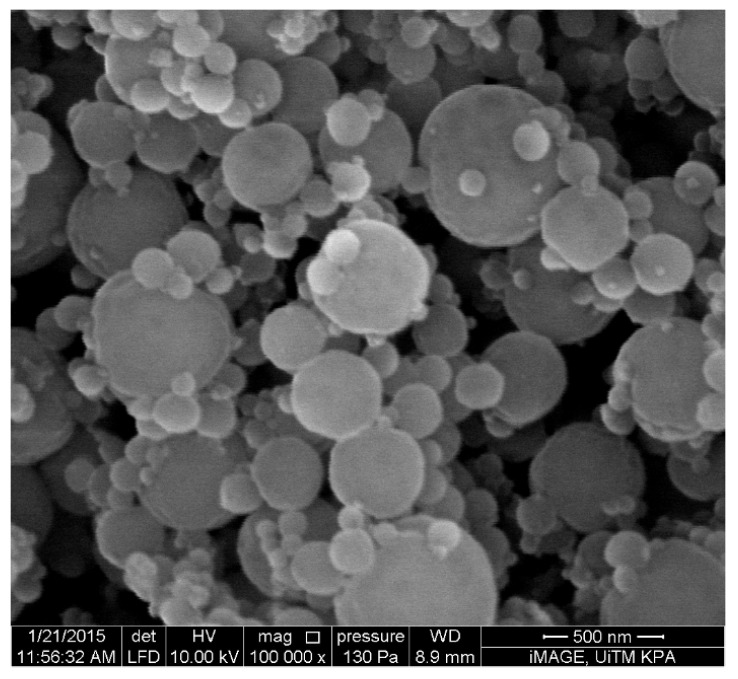
Scanning electron microscopic image of chitosan nanoparticles.

The chitosan nanoparticles had an average particle size of 684.00 ± 17.61 nm with a zeta potential of 27.86 ± 1.27 mV. They were spherical with a circularity value of 0.90 ± 0.23, a surface roughness *R_a_* of 0.38 ± 0.00 nm and a crystallinity index of 19.40 ± 2.23 % (Figure 3).

### 3.3. Nanoparticle–Microparticle Blend

**Figure 4:**
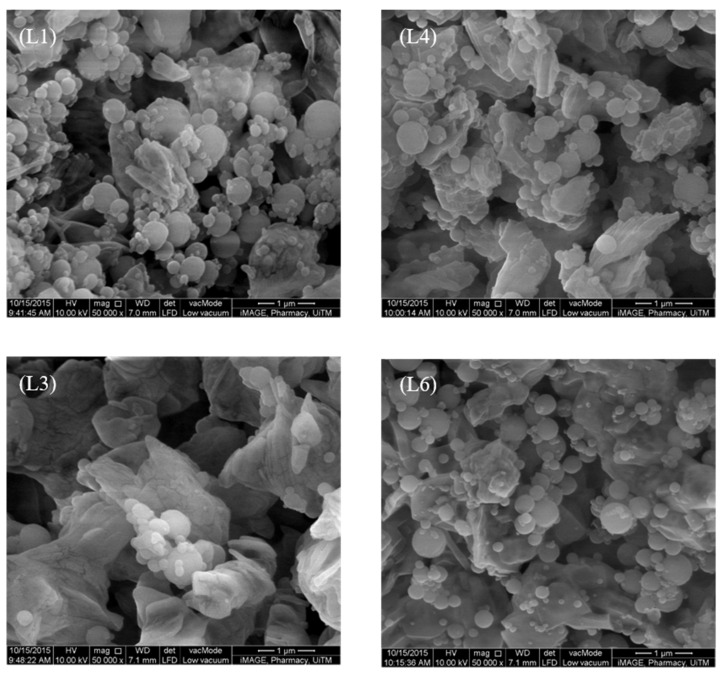
SEM micrographs of chitosan nanoparticle–lactose–PEG microparticle blends.

L2 and L5 were excluded from the inhalation study due to their high theoretical aerodynamic diameters of 7.37 ± 0.01 and 6.69 ± 0.04 µm respectively as inferred by their *d*_50_ and density values in preliminary trials. Figure 4 showed the distribution profiles of chitosan nanoparticles over lactose-PEG microparticles L1, L3, L4 and L6. Their aerosolization and inhalation profiles are summarized in Table 3.

Chitosan nanoparticles alone without blending with lactose-PEG 3000 microparticles exhibited poor inhalation performances (PD = 58.12 ± 23.30%; PI = 27.27 ± 12.62%). The inhalation performance is improved when the chitosan nanoparticles were blended with lactose-PEG 3000 microparticles (Table 3). Larger lactose-PEG microparticles (*d*_50_) with smaller specific surface areas brought about a higher respirable fraction of chitosan at the peripheral lung levels (Table 4). A reduction in particle size with an increase in the specific surface area of lactose-PEG microparticles increased its capacity to accommodate more nanoparticles. The mass formed would have a higher chance to deposit on higher stages due to their large mass and thus have reduced amount of nanoparticles reaching the lower stages. The circularity and surface roughness which theoretically had a strong bearing on a specific surface area appeared to play no critical role.

### 3.4. FTIR Study

The specific surface area and *d*_50_ of lactose-PEG microparticles exerted the largest effect on deposition manner of the chitosan nanoparticles (Table 4). L3 had the highest specific surface area with the lowest *d*_50_ values, whereas the opposite was applicable for L6. L3 and L6 were adopted to examine the attachment/detachment behavior of chitosan nanoparticles in an inhaled powder blend. FTIR spectra of the test powder blend collected at each stage were compared against the reference spectra, which constituted of lactose-PEG microparticles, chitosan nanoparticles, and their blend at a weight ratio of 9:1.

The changes in the correlation between the FTIR spectra of the test powder blend and the reference powder reflected the changes in the component ratio in the test powder blend. They did not necessarily reflect the changes in the amount of its components.

With reference to L3 and L6, marked correlation changes between spectra took place after stage 2 to 3 (Table 5). The spectra of the test powder blend collected between stage 0 and 3 were highly correlated to those of reference powder blend and lactose-PEG 3000 microparticles, the main component in a powder blend. At stages 0 to 3 generally, the chitosan nanoparticles were admixed with the lactose particles. At stages 4, 5 and 6, the weight ratio of chitosan nanoparticles to lactose-PEG 3000 microparticles appeared to be higher. At stage 7, the primary component in the test powder blend was chitosan nanoparticles. The test powder blend at stage 7 exhibited a sharp reduction in lactose-PEG 3000 microparticle fraction.

## 4. Conclusions

An inhalable microparticulate carrier can be used successfully to deliver nanoparticles in a form of coarse carrier-free dry powder inhaler. The specific surface area of the small microparticle carriers significantly affects the nanoparticle deposition on the airway. Larger microparticle carriers enhance the peripheral lung deposition of nanoparticles. They are characterized by a reduced specific surface area, which in turn facilitates the detachment of nanoparticles from the microparticle carrier and into the lower lung regions during inhalation. Unlike the blend of large carriers and nanoparticles [36], the inhalation efficiency of nanoparticles does not depend on shape and crystallinity of the microparticle carrier. Future studies shall focus on therapeutic loaded nanocarriers and their inhalational profiling through the aid of microparticle carriers in accordance with the compendial guidelines, and will examine the industry-relevance of microparticle carriers and its blend with nanocarriers. The therapeutic loading into nanoparticles is expected to be accompanied by a variation in their size, zeta potential and morphological characteristics. The critical inhalational quality attributes of microparticle carriers of a physical blend with the nanoparticles theoretically would remain unchanged. The required scale of these quality attributes, such as the magnitude of size and specific surface area, may however differ as a function of the physical characteristics of the therapeutic loaded nanoparticles. Further optimization of the particulate quality attributes is deemed necessary with reference to an in vivo application where the biological lung is characterized by a more complex anatomical structure and an electrostatic nature that has a strong bearing on particle flow and mucosal adhesion [37].

## Figures and Tables

**Table 1:** Formulation and process parameters of lactose-PEG microparticles.

Formulation/Processing Condition	L1	L2	L3	L4	L5	L6
Lactose concentration (%w/w)	2	2	2	2	2	2
PEG concentration (%w/w)	2.5	2.5	2.5	2.5	2.5	2.5
PEG molecular weight (g/mol)	400	1000	3000	400	1000	3000
Ethanol concentration (%w/w)	0	0	0	50	50	50
Feeding rate (mL/min)	2	2	2	2	2	2
Inlet temperature (°C)	70	70	70	70	70	70
Outlet temperature (°C)	26	25.6	27.5	26.9	27.3	27.6
Atomizing pressure (bar)	5.5	5.5	5.5	5.5	5.5	5.5

**Table 2:** Physicochemical attributes of lactose-PEG microparticles.

Particle Characteristic	L1	L2	L3	L4	L5	L6
Crystallinity (Peak area_2*θ*=19.98__°_)	888.27 ± 3.36	705.57 ± 14.10	508.73 ± 11.98	612.90 ± 17.90	764.03 ± 2.00	618.53 ± 13.48
*pb* (g/cm^3^)	0.150 ± 0.002	0.119 ± 0.001	0.102 ± 0.002	0.112 ± 0.003	0.091 ± 0.002	0.113 ± 0.002
*pt* (g/cm^3^)	0.283 ± 0.005	0.310 ± 0.001	0.212 ± 0.003	0.313 ± 0.002	0.252 ± 0.003	0.271 ± 0.002
Carr’s Index (%)	46.98 ± 1.67	61.51 ± 0.22	51.91 ± 0.13	64.25 ± 1.07	63.99 ± 0.52	58.47 ± 0.80
Hausner Ratio	1.89 ± 0.06	2.60 ± 0.02	2.08 ± 0.01	2.80 ± 0.08	2.78 ± 0.04	2.41 ± 0.05
Circ	0.12 ± 0.01	0.14 ± 0.02	0.16 ± 0.06	0.22 ± 0.02	0.27 ± 0.02	0.16 ± 0.02
Ra (nm)	129.73 ± 7.62	75.19 ± 1.47	127.21 ± 1.11	114.70 ± 17.83	86.24 ± 2.91	122.84 ± 1.25
Specific surface area (m^2^/g)	1.49 ± 0.08	1.24 ± 0.02	1.90 ± 0.01	1.55 ± 0.04	0.99 ± 0.02	1.31 ± 0.20
D_[4,3]_ (µm)	23.09 ± 0.64	19.23 ± 0.14	20.61 ± 1.04	15.53 ± 1.54	18.11 ± 3.44	20.74 ± 1.62
D_[3,2]_ (µm)	4.03 ± 0.25	4.84 ± 0.07	3.15 ± 0.02	3.87 ± 0.20	6.07 ± 0.14	4.62 ± 0.69
Span	6.57 ± 0.19	3.20 ± 0.04	9.76 ± 0.15	4.08 ± 0.27	2.05 ± 0.07	4.20 ± 0.35
d_10_ (µm)	1.72 ± 0.06	2.62 ± 0.06	1.58 ± 0.01	1.72 ± 0.04	4.02 ± 0.10	1.91 ± 0.18
d_50_ (µm)	9.33 ± 1.01	13.23 ± 0.18	5.43 ± 0.10	8.30 ± 0.27	13.32 ± 0.15	10.44 ± 0.48
d_90_ (µm)	63.03 ± 4.79	43.60 ± 1.14	54.60 ± 1.70	35.59 ± 1.86	31.30 ± 1.26	45.89 ± 5.85

*pb*: bulk density; *pt:* tapped density; Ra: surface roughness; D_[4,3]_: volume weighted mean diameter; D_[3,2]_: surface area weighted mean diameter; d_10_: particle diameter corresponding to 10% undersized fraction; d_50_: particle diameter corresponding to 50% undersized fraction; d_90_: particle diameter corresponding to 90% undersized fraction.

**Table 3:** Aerosolization and inhalation profiles of chitosan nanoparticles admixed with lactose-PEG microparticles L1, L3, L4 and L6.

	L1	L3	L4	L6
MMAD (µm)	2.95 ± 0.17	4.30 ± 0.85	3.90 ± 0.92	3.16 ± 0.30
GSD	3.22 ± 0.60	5.10 ± 0.35	3.74 ± 0.19	3.18 ± 0.40
TD mg	15	15	15	15
ED mg	11.81 ± 1.39	12.40 ± 1.80	12.81 ± 1.65	14.42 ± 0.49
DD mg	4.38 ± 0.29	6.70 ± 1.30	6.52 ± 1.33	7.87 ± 0.54
PD (%)	78.72 ± 9.30	82.80 ± 12.00	85.38 ± 10.99	96.12 ± 3.24
PI (%)	29.20 ± 1.92	44.40 ± 8.80	43.43 ± 8.84	52.44 ± 3.62
Particles < 4.5 µm				
FPD (mg)	3.51 ± 0.07	4.60 ± 1.20	4.57 ± 0.73	6.27 ± 0.59
FPF (%)	29.95 ± 2.96	36.96 ± 4.70	35.58 ± 1.22	43.47 ± 3.25
RF (%)	80.40 ± 6.34	69 ± 3.90	70.63 ± 4.20	79.66 ± 2.86
Particles < 3.6 µm				
FPD (mg)	2.21 ± 0.27	2.78 ± 0.85	2.98 ± 0.24	4.02 ± 0.52
FPF (%)	18.93 ± 3.36	22.14 ± 4.30	23.56 ± 3.79	27.81 ± 2.92
RF (%)	50.43 ± 3.08	41.23 ± 5.10	47.12 ± 10.63	50.94 ± 3.67
0.5 µm < Particles < 3.6 µm				
FPD (mg)	1.79 ± 0.128	1.98 ± 0.61	2.58 ± 0.22	3.58 ± 0.55
FPF (%)	15.39 ± 2.74	15.75 ± 3.08	20.44 ± 3.72	24.77 ± 3.32
RF (%)	40.97 ± 1.97	29.31 ± 3.70	40.94 ± 10.27	45.32 ± 4.09

MMAD: mass median aerodynamic diameter; GSD: geometric standard deviation; TD: total dose; ED: emitted dose; DD: deposited dose; PD: percent dispersed; PI: percent inhaled; FPD: fine particle dose; FPF: fine particle fraction; RF: respirable fraction.

**Table 4:** Pearson correlation of fine particle and respirable fractions of chitosan nanoparticles with physicochemical characteristics of lactose-PEG microparticles L1, L3, L4 and L6.

		r	*p*		r	*p*		r	*p*
	**FPF_<4.5_**			**FPF_<3.6_**			**_0.5<_FPF_<3.6_**		
Crystallinity (Peak area_2*θ=*19.98__°_)		−0.670	0.330		−0.571	0.429		−0.296	0.704
*pb* (g/cm^3^)		−0.701	0.299		−0.620	0.380		−0.362	0.638
*pt* (g/cm^3^)		−0.205	0.795		0.055	0.945		0.372	0.628
Carr’s Index (%)		0.532	0.468		0.684	0.316		0.720	0.280
Hausner Ratio		0.436	0.564		0.606	0.394		0.669	0.331
Circ		0.301	0.699		0.426	0.574		0.418	0.582
Ra (nm)		−0.324	0.676		−0.504	0.496		−0.583	0.417
Specific surface area (m^2^/g)		−0.272	0.728		−0.446	0.554		−0.703	0.297
D_[4,3]_ (µm)		−0.202	0.798		−0.342	0.658		−0.361	0.639
D_[3,2]_ (µm)		0.380	0.620		0.530	0.470		0.753	0.247
Span		−0.288	0.712		−0.522	0.478		−0.776	0.224
d_10_ (µm)		0.568	0.432		0.693	0.307		0.860	0.140
d_50_ (µm)		0.192	0.808		0.361	0.639		0.629	0.371
d_90_ (µm)		−0.503	0.497		−0.652	0.348		−0.680	0.320
	**RF_<4.5_**			**RF_<3.6_**			**_0.5<_RF_<3.6_**		
Crystallinity (Peak area*_2θ_*_=19.98°_)		0.744	0.256		0.686	0.314		0.468	0.532
*pb* (g/cm^3^)		0.724	0.276		0.635	0.365		0.401	0.599
*pt* (g/cm^3^)		0.311	0.689		0.685	0.315		0.777	0.223
Carr’s Index (%)		−0.363	0.637		0.044	0.956		0.346	0.654
Hausner Ratio		−0.362	0.638		0.067	0.933		0.363	0.637
Circ		−0.648	0.352		−0.251	0.749		0.041	0.959
R_a_ (nm)		0.398	0.602		−0.044	0.956		−0.331	0.669
Specific surface area (m^2^/g)		−0.803	0.197		−0.964 *	0.036		−0.989 *	0.011
D_[4,3]_ (µm)		0.634	0.366		0.222	0.778		−0.058	0.942
D_[3,2]_ (µm)		0.808	0.192		0.936	0.064		0.963 *	0.037
Span		−0.408	0.592		−0.765	0.235		−0.920	0.080
d_10_ (µm)		0.715	0.285		0.840	0.160		0.908	0.092
d_50_ (µm)		0.855	0.145		0.986 *	0.014		0.979 *	0.021
d_90_ (µm)		0.412	0.588		0.004	0.996		−0.300	0.700

* Correlation is significant at the 0.05 level (2-tailed). *pb*: bulk density; *pt*: tapped density; Ra: surface roughness; D_[4,3]_: volume weighted mean diameter; D_[3,2]_: surface area weighted mean diameter; d_10_: particle diameter corresponding to 10% undersized fraction; d_50_: particle diameter corresponding to 50% undersized fraction; d_90_: particle diameter corresponding to 90% undersized fraction; FPF: fine particle fraction; RF: respirable fraction.

**Table 5:** FTIR spectra correlations of L3 and L6 admixed chitosan nanoparticles against chitosan nanoparticles, lactose-PEG 3000 microparticles and their blend.

		L3			L6	
Stage	Chitosan Nanoparticles	Lactose-PEG 3000 Microparticles	Blend	Chitosan Nanoparticles	Lactose-PEG 3000 Microparticles	Blend
0	0.5316 ± 0.013	0.9815 ± 0.007	0.9888 ± 0.005	0.5280 ± 0.009	0.9809 ± 0.005	0.9868 ± 0.003
1	0.5338 ± 0.009	0.9578 ± 0.006	0.9838 ± 0.007	0.5530 ± 0.007	0.9228 ± 0.006	0.9365 ± 0.006
2	0.5279 ± 0.008	0.9482 ± 0.008	0.9882 ± 0.004	0.6029 ± 0.013	0.8754 ± 0.022	0.8963 ± 0.020
3	0.5755 ± 0.010	0.9545 ± 0.003	0.9832 ± 0.001	0.6064 ± 0.006	0.7437 ± 0.014	0.7743 ± 0.016
4	0.7013 ± 0.046	0.8406 ± 0.031	0.8228 ± 0.030	0.6960 ± 0.001	0.7638 ± 0.019	0.7936 ± 0.019
5	0.7093 ± 0.012	0.9120 ± 0.001	0.8861 ± 0.004	0.6879 ± 0.010	0.8523 ± 0.016	0.8727 ± 0.011
6	0.6527 ± 0.007	0.9186 ± 0.004	0.8931 ± 0.005	0.6783 ± 0.059	0.7688 ± 0.054	0.7668 ± 0.060
7	0.9281 ± 0.005	0.4965 ± 0.010	0.5546 ± 0.011	0.9203 ± 0.008	0.3968 ± 0.007	0.4392 ± 0.002

## Data Availability

The data presented in this study are available on request from the corresponding author.

## Abbreviations

FTIR: Fourier transform infrared spectroscopy; PEG: polyethylene glycol; SEM: scanning electron microscope; XRPD: X-ray powder diffractometer.
